# A comparative study of spiritual health in HIV-positive patients and healthy individuals

**DOI:** 10.1038/s41598-026-42631-8

**Published:** 2026-04-19

**Authors:** Maryam Ansari Aval, Fatemeh Ayoubi, Mohammad Khajedaluee, Maliheh Dadgarmoghaddam

**Affiliations:** 1 Community Medicine Department, Faculty of Medicine, Mashhad University of Medical Sciences, Mashhad, Iran; 2 Faculty of Medicine, Mashhad University of Medical Sciences, Mashhad, Iran

**Keywords:** Existential well-being, Human immunodeficiency virus, Spiritual well-being, Diseases, Health care, Medical research, Risk factors

## Abstract

Given the role and importance of spiritual well-being in individuals living with AIDS and the role of psychological interventions on the spiritual components of these patients, the present study was conducted to study and examine the spiritual well-being of HIV-positive patients and compare it with healthy individuals. This was a descriptive-analytical cross-sectional study conducted in Mashhad (a metropolitan area in northeast of Iran) in 2021. A census method was employed for the research, involving HIV-positive patients and a random sample of healthy individuals visiting the Comprehensive Health Center, who participated after giving informed consent. The demographic form and the Spiritual Well-Being Questionnaire (SWBQ) were completed by the patients. The statistical analysis was performed using SPSS software (version 11.5), and the significance level was considered at *p* < 0.05. A total of 201 participants entered the study with a mean age of 38.29 ± 10.53 years, including 102 HIV-positive patients and 99 healthy individuals. The total spiritual well-being score in the HIV group was 77 (57–84), while the healthy group scored 100 (88–107) (*P* < 0.001). Regression analysis showed that occupation and HIV infection were two significant variables in the model for prediction the total spiritual well-being score. The total score of AIDS patients on the Spiritual Well-Being Questionnaire was significantly lower than that of healthy individuals. It is recommended that the health system authorities take action to increase the spiritual well-being of HIV-positive patients.

## Introduction

The World Health Organization (WHO)defines health as having physical, cognitive, and social functioning without diseases and disabilities. From a holistic perspective, health is considered a feeling of well-being across four dimensions: physical, mental, social, and spiritual^1^. In various declarations and statements of the WHO, the role of spiritual health and its elements in promoting health and achieving sustainable development has been emphasized^2–5^.

Numerous scientific evidence today confirms the impact of individuals’ spirituality and beliefs on their health and quality of life^6^, maintaining and enhancing self-esteem, creating a sense of purpose and meaning in life, psychological well-being, hope, increased adaptability and coping ability, and a sense of calmness and strength in dealing with the crisis of illness. Patients with spiritual inclinations are often able to find meaning and purpose even during illness, enabling them to cope more effectively with pain and suffering and to navigate through challenging times. Patients with spiritual tendencies, despite the crisis of illness, are able to find meaning and purpose in their lives and are more successful in coping with the pain and suffering associated with disease and emerging from the crisis^7^.

The provision of treatment and medical care should be in accordance with the beliefs of the people in the community. For this reason, it is recommended that the concept of spiritual health be explained differently in distinct societies, taking into account their specific culture and beliefs. For this reason, contemporary science defines spiritual health in two dimensions: religious health and existential health.Religious health focuses on the quality and nature of an individual’s perception of health in their spiritual life when they are connected to a higher power (God). Existential health refers to the individual’s adaptation to people, self, and the environment, and it discusses the individual’s social and psychological concerns^8^. Some believe that without spiritual health, achieving maximum performance in other dimensions of health is impossible, and disruption in spiritual health is a factor in mental disturbances, anxiety, depression, and loss of meaning in life and is an obstacle to achieving high levels of quality of life^9,10^.

Acquired Immune Deficiency Syndrome (AIDS )is a chronic and progressive disease that has created complex health problems, affecting the quality of life. Patients with this disease need spiritual support to increase their spiritual health^11^. The HIV/AIDS phenomenon is one of the most important health, social, and economic problems in many countries around the world, which is growing in scope and dimensions day by day. In such circumstances, this phenomenon should be considered a crisis in the health of human societies^12^. Studies show that the most of the people infected with HIV live in the Third World, especially in African countries, and the prevalence of this phenomenon is increasing more dramatically in these areas^13^.

Firers and Machin state that HIV/AIDS patients in their daily lives must be able to cope with complex physical, psychological, and spiritual problems. The complexity of the problems ahead of them affects their quality of life^14^. Dalmeida et al. proposed a more effective approach to treatment using spirituality. This approach involves psychological counseling and helps health professionals in the treatment of patients. Spirituality and religion play significant and positive roles in the treatment of AIDS^15^.

Iran has also been exposed to this global phenomenon in recent years and is faced with number of people infected with the immunodeficiency virus and suffering from AIDS^16^. This disease, due to its particular nature, seriously impacts all aspects of the existence and life of those infected, as well as the health of the community. However, the diagnosis of AIDS and being infected with it places the individual in an ambiguous and difficult situation, confronting them with the challenging question of what meaning and purpose their lives will hold in the long term with this major problem.

Is it possible to argue that encountering challenging and threatening situations, such as contracting AIDS, may provide individuals with an opportunity to reflect more deeply on their lives and give them meaning? According to the available information, the answers to these questions still remain ambiguous and require further study. Additionally, all healthcare workers must be aware of the concept and living conditions with this phenomenon, as well as the role of religious, spiritual, and human beliefs, convictions, and values in the lives of those infected. They should also have a proper understanding of their experiences regarding the mentioned important issues^17^.

According to the researchers’ opinions and considering the points mentioned, conducting more extensive research and gaining a deeper understanding of the everyday life experiences of HIV-infected patients, as well as the role of spiritual beliefs and ideas, can provide a suitable basis for the performance of the healthcare team. Given the above, this study was conducted with the aim of examining spiritual health in HIV-positive patients and comparing it with healthy individuals.

## Method

This study was a cross-sectional, two-group research carried out at Mashhad University of Medical Sciences(Razi Khorasan, Mashhad, Iran) in 2021. The tools used in the study included a demographic form and a spiritual health questionnaire. The participants consisted of HIV-positive patients as the case group and non-infected individuals as the control group.

The census method was used for this study. The required sample size for this study was estimated to be 100 based on the number of active records at the center. These individuals were selected from Health Center No. 1 in Mashhad, which is responsible for providing counseling on behavioral diseases to individuals who are covered by the Health Center(At 2021). Additionally, during visits to the Mashhad Comprehensive Health Center, a random sample of 100 healthy individuals were interviewed within a one-month period.

After visiting the centers, the questionnaires were completed. The interviews were conducted by trained interviewers who possessed effective communication skills for interacting with patients. Verbal consent was obtained from the participants, and they were allowed to refrain from completing the questionnaire if they did not wish to participate. All information related to the participants was kept confidential, and codes were used instead of names for recording.

The tools used in this study to gather the required data included demographic characteristics form and the Spiritual Well-Being Questionnaire (SWBQ), which consists of 20 standardized items rated on a Likert scale. This questionnaire developed by Palutzian and Ellison (1998)^18^. It’s divided into two parts: religious well-being and existential well-being, with each part having 10 items. The odd-numbered items evaluate religious well-being, and the even-numbered items assess existential well-being. This scale is a questionnaire containing 20 statements, with responses based on a 6-point Likert scale (ranging from strongly agree to strongly disagree). The scale is divided into two groups: religious health and existential health, each consisting of 10 statements, with a score range of 10–60. The statements reflect individual religious health and the paired statements reflect existential health. The total score for spiritual health is the sum of the scores from these two subgroups, ranging from 20 to 120. In statements with a positive verb, the response “strongly agree” receives a score of 6, while “strongly disagree” receives a score of 1. For statements with a negative verb, the response “strongly agree” receives a score of 1, and “strongly disagree” receives a score of 6. The total scores can be categorized as follows: Low spiritual health: 20–40, Moderate spiritual health: 41–99, High spiritual health: 100–120^18^.

The validity and reliability of this questionnaire have been confirmed in Iran. Higher scores indicate greater spiritual well-being^19^.

The description of the population was carried out in the form of central and dispersion indices and frequency tables. The chi-square test was used to compare qualitative variables between two groups. The Mann-Whitney U test was used to compare the quantitative variables based on the distribution of the variable. To predict the total score of the questionnaire, linear regression analysis using the Enter method was employed. Significant variables in the univariate analysis were entered into the model (P < = 0.05). In all calculations, the level of significance was set at less than 0.05.

### Ethical considerations

All stages of the study were conducted based on the ethical principles of the Helsinki Declaration. Verbal consent was obtained from the participants before entering the study, and they could refuse to complete the questionnaire if they did not wish to. This protocol was approved by the Biomedical Research Ethics Committee part of Mashhad University of Medical Sciences with Ethical approval reference number IR.MUMS.MEDICAL.REC.1399.632.

### Findings

A total of 201 participants were included in the study with a mean age of 38.29 ± 10.53 years, of whom 102 were HIV-positive patients and 99 were healthy individuals. Table 1 presents the demographic characteristics of the enrolled participants. The status of demographic and background variables was compared between the two groups. The Chi-square test showed that the two groups differed significantly in terms of occupational status (*P* < 0.001), marital status (*P* < 0.001), income level (*P* < 0.001), education level (*P* < 0.001) and gender (*P* = 0.514) .However place of residence (*P* = 0.05) were similar between the two groups. Additionally, the median (interquartile range) age was 41 (36–48) years in the HIV group and 35 (25.75-40) years in the healthy group, and the Mann-Whitney test showed significant difference in age between the two groups (*P* < 0.001).

**Table 1:** Characteristics of participants in the two groups.

Characteristic		Frequency	%	Frequency	%	pvalue
		HIV+		Control		
Gender	Male	75	73.5	50	51	< 0.001
	Female	27	26.5	48	49	
Occupation	Unemployed	41	40.2	9	9.4	< 0.001
	Employee	1	1	18	18.8	
	Nongovernmental	50	49	23	24	
	Student	0	0	19	19.8	
	Housewife	10	9.8	27	28.1	
Place of residence	Mashhad	100	98	90	91.8	= 0.056
	Town	2	2	8	8.2	
Marital status	Married	44	43.1	70	72.2	< 0.001
	Single	31	30.4	23	23.7	
	Divorced	22	21.6	3	3.1	
	Widowed	5	4.9	1	1	
Income level	Low	85	83.3	35	35.7	< 0.001
	Middle	17	16.7	61	62.2	
	High	0	0	2	2	
Education	Illiterate	6	5.9	3	3.1	< 0.001
	Under diploma	80	78.4	20	20.8	
	Diploma	15	14.7	47	49	
	Bachelor and above	1	1	26	27.1	

Table  2 compares the participants’ responses to each of the questionnaire items between the HIV-positive and healthy groups. As can be observed, the responses to all 20 items differed significantly between the HIV and healthy groups (*P* < 0.005). The most common mode of transmission was sexual contact, occurring in 52 patients (52.5%), followed by blood contact in 46 patients (46.5%). Vertical transmission from mother to child showed the lowest percentage (1%).

**Table 2:** Comparison of participants’ responses in the two groups to each of the questionnaire items.

Item	Healthy group Median (IQR)	HIV group Median (IQR)	*P* value^*^
I do not feel much satisfied with praying and being alone with God	6 (5–6)	4 (3–4)	< 0.001
I do not know who I am, where I came from, and where I will go	5 (4–6)	4 (3–4)	< 0.001
I believe that God loves me and watches over me at all times	6 (5–6)	4 (3–4)	< 0.001
I feel that life is a positive and pleasant experience	5 (5–6)	4 (3–4)	< 0.001
I believe that God has no role in my life	6 (5–6)	4 (3–5)	< 0.001
I feel that I have an uncertain future	5 (3–6)	4 (3–4)	< 0.001
I have a special spiritual connection with God	5 (4–6)	3 (2–4)	< 0.001
I have reached the pinnacle of life and feel completely satisfied	4 (3–5)	3 (2–4)	< 0.001
I feel that I am not supported and empowered by God	6 (5–6)	4 (3–5)	< 0.001
I feel good about the path of life that lies ahead of me	5 (4–6)	4 (3–4)	< 0.001
I believe that God does not pay attention to my problems	6 (5–6)	4 (3–4)	< 0.001
I do not get enough enjoyment from my life	5 (3–6)	4 (3–4)	0.004
I do not have a satisfying personal relationship with God	5 (4–6)	4 (3–4)	< 0.001
I feel good about my future	5 (4–6)	4 (3–4)	< 0.001
My relationship with God helps me not feel lonely	5 (5–6)	4 (3–4)	< 0.001
I feel that life is full of suffering and distress	4 (3–6)	4 (3-4.25)	< 0.001
When I have a close relationship with God, I feel a sense of perfection	6 (5–6)	4 (3–4)	< 0.001
Life has little meaning and purpose	6 (4–6)	4 (3–5)	< 0.001
My relationship with God plays a role in my sense of well-being	6 (5–6)	4 (3–4)	< 0.001
I believe that there is a special purpose for my existence	5 (4–6)	4 (3–4)	< 0.001

*Mann-Whitney test.

Finally, the scores for participants in both groups were computed based on the questionnaire, as presented in Table 3. The Mann-Whitney statistical test indicated significant differences in scores between the two groups in each of the existential and religious sections, as well as in total (*P* < 0.001).

**Table 3:** Comparison of spiritual and religious health scores and total scores between HIV-positive patients and healthy individuals.

Characteristic	HIV group	Healthy group	*P* value*
Total score, median (interquartile range)	77 (57.0–84.0)	100 (88–107)	< 0.001
Spiritual health score, median (interquartile range)	38 (29.0–42.0)	47 (42.0–51.0)	< 0.001
Religious health score, median (interquartile range)	39 (28.0–41.0)	54 (47.0–58.0)	< 0.001

*Mann-Whitney test.

Table 4 presents the scores for the religious domain, existential domain, and the total score in the healthy and HIV-positive groups. It is evident that there are significant differences in the scores for the religious and existential domains, as well as the total score, between the two groups.

**Table 4:** Comparison of dimensions of spiritual and religious health between HIV-positive patients and healthy individuals.

Characteristic		HIV group	Healthy group	*P* value*
Religious health score	Low	2 (2.0)	1 (1.0)	< 0.001
	Middle	97 (95.1)	36 (36.4)	
	High	3 (2.9)	62 (62.6)	
Spiritual health score	Low	3 (2.9)	1 (1.0)	< 0.001
	Middle	97 (95.1)	63 (63.6)	
	High	2 (2.0)	35 (35.4)	
Total score	Low	2 (2.0)	0 (0.0)	< 0.001
	Middle	97 (95.1)	47 (47.5)	
	High	3 (2.9)	52 (52.5)	

*Mann-Whitney test.

Comparing scores between the two genders showed that the total spiritual health score was higher in women than in men(89(76–103)Vs 83(60–96)), and this difference was statistically significant(p value < 0.02).

To predict the total score of the questionnaire, linear regression analysis using the Enter method was employed. Significant variables in the univariate analysis were entered into the model (*P* < 0.05). As can be observed, occupation and HIV-positive status were the two significant variables in the model (Table 5).

**Table 5:** Linear regression analysis for prediction of total score.

Variables	Unstandardized coefficients		Standardized coefficients	t	Sig.	95.0% confidence interval for B	
	B	Std. error	Beta			Lower bound	Upper bound
(Constant)	28.932	10.994		2.632	0.009	7.240	50.623
Age	− 0.002	0.123	− 0.001	− 0.017	0.986	− 0.245	0.241
Gender	0.641	2.720	0.015	0.236	0.814	-4.726	6.009
Occupation	2.041	0.953	0.142	2.143	0.033	0.162	3.921
Place of residence	5.069	5.152	0.056	0.984	0.326	-5.096	15.235
Marital status	1.486	1.696	0.053	0.876	0.382	-1.860	4.832
Income level	4.544	2.629	0.116	1.729	0.086	− 0.643	9.730
Education	1.623	1.862	0.062	0.872	0.385	-2.051	5.296
Group(HIV positive code 0,healthy group code1)	19.619	3.117	0.489	6.294	0.001	13.469	25.768
Dependent variable: total score.							

R Model: 0.66.

Model: Enter.

Core assumptions (linearity, homoscedasticity, normality of residuals) were checked.

## Discussion

The results of our study showed that the total scores of HIV-positive patients in both the religious health and existential health parts of the questionnaires were significantly lower than those of healthy individuals. Additionally, the analysis of responses from participants with AIDS compared to other individuals revealed significant differences across all questionnaire items.

In the positively worded items related to existential health, such as “I feel that life is a positive and pleasant experience,” “I have reached the pinnacle of life and feel completely satisfied,” “I feel good about the path of life that lies ahead of me,” “I feel good about my future,” and “I believe there is a specific purpose for my existence,” the proportion of agreeing and strongly agreeing responses was significantly lower in the group of HIV-positive patients than in the other group.

In contrast, in the negatively worded items related to existential health, such as “I do not know who I am, where I came from, and where I’m going,” “I feel I have an uncertain future,” and “I do not enjoy my life enough,” the HIV-positive patients had fewer disagreeing and strongly disagreeing responses compared to the other group.

The questions related to religious health, which were the odd-numbered items in the questionnaire, reflected a situation similar to that of the existential health items. In the positively worded religious health items, such as “I believe that God loves me and watches over me at all times,” “I have a special spiritual relationship with God,” “My relationship with God helps me not feel lonely,” “When I have a close relationship with God, I feel fulfilled,” and “My relationship with God plays a role in my sense of well-being,” the agreeing and strongly agreeing responses of the healthy participants were significantly higher than those of the HIV-positive patients.

In religious health items with negative meanings, such as “I do not feel much satisfied with prayer and solitude with God,” “I believe that God has no role in my life,” “I feel that I am not supported and empowered by God,” and “I do not have a satisfying personal relationship with God,” the disagreeing and strongly disagreeing responses of the healthy participants were higher than those of the HIV-positive individuals.

In a study conducted in 2017 by Ebrahimbabaie et al., the authors compared the health-promoting lifestyle between individuals with and without HIV. The results showed that there was a significant difference between the two groups in terms of lifestyle variables. Specifically, the levels of health responsibility and physical activity were lower in the HIV-positive group compared to the non-HIV group. However, no significant differences were observed between the two groups in the components of nutrition, interpersonal relationships, spiritual growth, and stress management^20^.

Our study results are different from those reported by Ebrahimbabaie et al. In our study, the spiritual health of patients with HIV was lower than that of other individuals, while in the study by Babaei et al., the spiritual growth of the two groups was found to be similar. However, it should be noted that due to the difference in the tools used in the two studies to assess the spiritual status of the participants, a precise comparison between the obtained results cannot be made.

In another study conducted in 2010 by Cuevas et al., the authors examined spirituality and religious beliefs in patients with AIDS and healthy individuals. A total of 201 people participated in the study, who were divided into four groups: elderly with HIV, healthy elderly, young with HIV, and healthy young. The results showed that there was no significant difference in the levels of spirituality and religious beliefs among the four study groups. However, among patients with HIV, individuals with greater spirituality had better social networks, better self-reported health, and fewer medical problems compared to those with lower spirituality^21^.

The results obtained from our study were inconsistent with those of a study conducted by Cuevas. In our research, patients with HIV had lower spirituality and existential well-being compared to individuals without HIV. The discrepancy in the results between the two studies may be attributed to the difference in the measurement tools used. The Cuevas study used the Ironson-Woods Spirituality/Religiousness Index to assess individuals’ spirituality, while our study employed different measurement tools. Additionally, the cultural and social differences between the study populations (Iran and the United States) may have contributed to the differing results.

Numerous studies in this field indicate that spirituality, focus on religious beliefs, and attention to God have led individuals to experience fewer issues and conflicts related to HIV/AIDS. Additionally, these factors serve as significant resources for addressing and managing the challenges they face in their lives^22,23^.

A significant potential explanatory factor for the lower spiritual well-being scores observed among patients living with HIV compared to the healthy control group in the Iranian context is the profound burden of HIV-related stigma. In settings like Iran, where socio-cultural norms are deeply interwoven with traditional religious values, HIV stigma is often exacerbated by moral judgments surrounding transmission routes, pervasive misconceptions about the disease, and a lack of public health literacy. This stigmatization can manifest as social rejection, discrimination, and internalized shame, leading to social isolation, diminished psychosocial support, and profound feelings of guilt and unworthiness. Such psychological and social sequelae directly conflict with the core dimensions of spiritual well-being, including a sense of community belonging, the capacity to find meaning and purpose in life, and the ability to maintain a positive connection with a higher power (God). When individuals face rejection or perceive themselves as deserving of blame due to their serostatus, their journey toward self-forgiveness, hope, and existential meaning—essential components of spiritual health—can be severely obstructed. Consequently, HIV-related stigma operates not merely as a psychosocial stressor but as a critical barrier to spiritual resilience and transcendence, potentially accounting for the compromised spiritual well-being in this population relative to their healthier counterparts^24–27^.

This study offers several notable strengths, including the use of a standardized, validated instrument to assess spiritual well-being, enhancing the comparability and reliability of our findings. Furthermore, employing multivariable regression analysis allowed us to statistically control for key demographic confounders, strengthening the internal validity of the observed association between HIV status and spiritual well-being. However, several limitations warrant consideration when interpreting the results. First, the cross-sectional design precludes any inference of causality or directionality in the relationship between HIV-related stigma and spiritual well-being. Second, despite statistical adjustments, the baseline demographic differences between the census-based patient group and the randomly selected control group indicate potential residual confounding. Finally, data were collected from a single referral center in Iran; thus, the findings may not be fully generalizable to all people living with HIV in other clinical or cultural settings. Future longitudinal studies with matched control groups and multi-center designs are needed to confirm these associations and explore their temporal dynamics.

## Conclusion

The results of our study showed that patients with HIV had lower religious and existential health status compared to healthy individuals, which is concerning. This indicates that these patients may require social and psychological follow-ups. The overall score of the questionnaire was also significantly lower in HIV patients than in healthy individuals. Further studies in this field involving different populations could also be beneficial.

## Data Availability

The data that support the findings of this study are available in Excel format, and so are not publicly available. Data are however available from the corresponding author upon reasonable request and with permission of Mashhad University of Medical Sciences.
